# Hyaluronic Acid as a Modern Approach in Anticancer Therapy-Review

**DOI:** 10.3390/ijms24010103

**Published:** 2022-12-21

**Authors:** Monika Michalczyk, Ewelina Humeniuk, Grzegorz Adamczuk, Agnieszka Korga-Plewko

**Affiliations:** Independent Medical Biology Unit, Medical University of Lublin, 20-090 Lublin, Poland

**Keywords:** hyaluronic acid, hyaluronic acid receptors, cancer, anticancer therapy

## Abstract

Hyaluronic acid (HA) is a linear polysaccharide and crucial component of the extracellular matrix (ECM), maintaining tissue hydration and tension. Moreover, HA contributes to embryonic development, healing, inflammation, and cancerogenesis. This review summarizes new research on the metabolism and interactions of HA with its binding proteins, known as hyaladherins (CD44, RHAMM), revealing the molecular basis for its distinct biological function in the development of cancer. The presence of HA on the surface of tumor cells is a sign of an adverse prognosis. The involvement of HA in malignancy has been extensively investigated using cancer-free naked mole rats as a model. The HA metabolic components are examined for their potential impact on promoting or inhibiting tumor formation, proliferation, invasion, and metastatic spread. High molecular weight HA is associated with homeostasis and protective action due to its ability to preserve tissue integrity. In contrast, low molecular weight HA indicates a pathological condition in the tissue and plays a role in pro-oncogenic activity. A systematic approach might uncover processes related to cancer growth, establish novel prognostic indicators, and identify potential targets for treatment action.

## 1. Introduction

Cancer is the second leading cause of death worldwide, after cardiovascular disorders [1]. Reports indicate that cancer-related deaths will continue to grow, reaching 13.1 million by 2030 [2,3,4,5]. For these reasons, effective oncological treatment is one of the most essential purposes of modern medicine. After decades of impressive efforts to discover the biology of cancer, the most challenging questions still arise in the context of cancer treatment [6]. In many cases, the poor efficacy and severe side effects of a variety of these therapeutic strategies remain an issue [7]. Hyaluronic acid (HA) plays a major role in aging, cellular senescence, cancer development and progression, and tissue homeostasis. The molecular weight of HA differs, which affects its physiological functions and processes. High molecular weight HA (HMW-HA) exhibits anti-inflammatory, anti-proliferative, and anti-angiogenic effects, and it also aids in wound healing [8]. In homeostasis, HA is present in almost all human tissues in the form of HMW-HA. Nevertheless, pathological conditions, including inflammation or cancerogenesis, reveal indications of increased HA fragmentation, resulting in a larger proportion of HA polymers with a lower molecular weight (LMW-HA) [9]. As a result, the effects of HA in the pathological environment are frequently connected with the polymer’s changing mass. Surprisingly, the molecular weight range of HA in different species varies [10]. Furthermore, the interaction of HA with its binding partners, the hyaladherins, such as CD44, is essential for sustaining tissue integrity and is likewise related to cancer [11]. The naked mole rat, a rodent species, possesses a special form of very high molecular weight (vHMW-HA), which is associated with the extraordinary cancer resistance and longevity of those animals [12]. There is a discrepancy between the high amounts of HA found in naked mole rats, which contribute to their resistance to cancer, and high levels of HA discovered in human neoplastic tissues, which suggest a carcinogenic process and poor prognosis [13]. To comprehend this essential aspect, it is necessary to explore the various sizes of HA that may exist in the human body. The purpose of this study is to conduct a review of the literature in which the importance of hyaluronic acid in cancer biology and anticancer therapy, as well as in cancer diagnostics, has been investigated and proved.

## 2. Basic Information about Hyaluronic Acid

**Figure 1:**
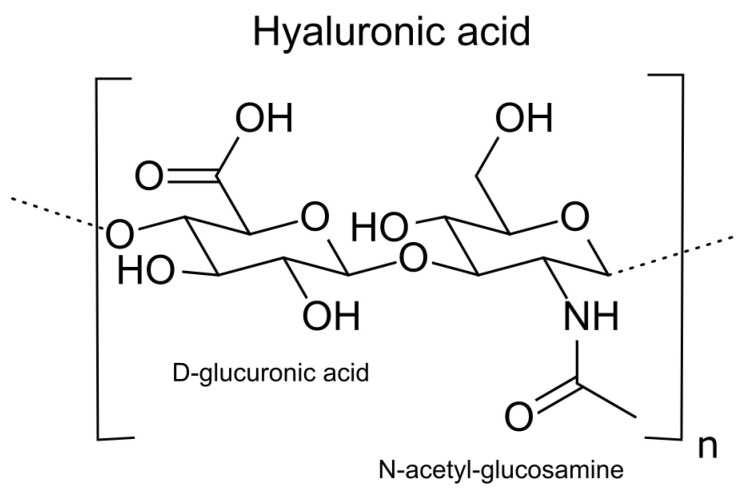
The structural formula of a disaccharide building block of the hyaluronic acid polymer composed of alternating D-glucuronic acid and And-acetyl-d-glucosamine units; n indicates the number of repeating units in a polymer molecule.

Hyaluronic acid (HA) is a polysaccharide with alternating units of D-glucuronic acid and N-acetyl-D-glucosamine linked by β (1 → 4) and β (1 → 3) glycosidic bonds (Figure 1). The carboxyl group of each D-glucuronic acid unit is usually dissociated, leading to a negatively charged bio-molecule at physiological pH values [14,15]. The interaction of HA with specific receptors on the cell surface determines its properties. It belongs to the group of non-protein, unsulfated glycosaminoglycans (GAG) [16,17]. As opposed to other GAGs, HA is synthesized in plasma membranes and then transported into the pericellular space [18]. In homeostasis, native HA consists of 2000–25,000 disaccharide units, 2–25 μm long. The molecular weight of HA is crucial for the properties of this acid and its effect on other cells and tissues. HMW-HA is linked with homeostasis and protective action. In contrast, LMW-HA indicates a pathological condition in the tissue [11,19].

HA naturally occurs in the extracellular matrix (ECM) in the human body in the form of sodium salt (sodium hyaluronate). It is present in the highest concentration in the skin and synovial fluid. HA is also a component of body fluids, epithelial and nervous tissues, and connective tissue. Additionally, HA builds the walls of blood vessels [20]. Fibroblasts, chondrocytes, and synoviocytes are responsible for the synthesis of HA [21]. The tissue microenvironment, as well as intercellular signaling factors, influences the synthesis and degradation of HA [22]. Many studies have confirmed the involvement of HA in the processes of wound healing, tissue structure, cell signaling, and tissue hydration [23]. Moreover, specific and non-specific interactions of HA contribute to embryonic development, differentiation, ECM organization, cell adhesion, inflammation, tissue regeneration, and organ structural stability [24].

Considerable alterations in the characteristics of ECM elements may be detected during tumor growth. The change in the phenotype of cancer cells from pre-invasive to invasive phenotype is related to substantial changes in the composition of the ECM in the tumor microenvironment. It leads to enhanced angiogenesis and inflammation, resulting in the formation of a mitogenic environment [25]. Malignant cells during interaction with HA may alter the ECM, while the matrix might influence tumor cell activity [10,26]. This HA-rich matrix may presumably contribute to cell motility, which is required for metastatic progression [27]. By altering the biomechanical properties of tumor tissue, HA encourages cellular separation during mitosis, forms a hydrated matrix, and boosts cellular mobility and proliferation [28].

## 3. Synthesis and Decomposition of Hyaluronic Acid

HA is synthesized by integral membrane proteins called hyaluronan synthases (HAS). These synthases are present in three types: HAS1, HAS2, and HAS3. They take part in extending HA by repeatedly adding D-glucuronic acid and N-acetyl-D-glucosamine to the resulting polysaccharide [29]. It is extruded by the ABC transporter across the cell membrane into the extracellular space [30].

HAS1 is the least active enzyme producing HA, and its role in health and disease remains relatively poorly understood [31]. HAS2 is particularly active during embryonic development, producing HMW-HA, which is important for facilitating the coordination of numerous essential cellular processes during early development. In turn, its level increases during pathological or stressful conditions, such as inflammation, septicemia, burns, shock, or in the case of severe wounds [32].

HAS3 is the most active enzyme. During tissue growth and repair, HAS3 generates considerable amounts of low molecular weight HA. Additionally, HAS3 is a crucial element of ECM and contributes to resistance to compressive tissue forces, tissue homeostasis, and articular joint surface hydration [33].

The action of these enzymes depends on the level of transcription, translation, and posttranslational modifications—for example, alternative splicing and epigenetic processes. HA arises as large, elongated, unbranched structures, but each enzyme contributes to the synthesis of different sizes of HA polymers. Thus far, several studies have reported that deficiency of these synthase enzymes leads to heart and vessel defects in mid-pregnancy, followed by embryo mortality [34]. Additionally, different cytokines and growth factors affect the synthesis efficiency. HAS1 expression is upregulated by transforming growth factor beta (TGF-β), while HAS3 is overexpressed by IL-1β and TNF-α. Moreover, various molecules and hormones have an impact on HA synthesis, e.g., phorbol esters (PMA), platelet-derived growth factors, and glucocorticoids that directly or indirectly boost or arrest HA synthesis [20].

Degradation of HA is possible by breaking it down by a family of enzymes called hyaluronidases. In humans, there are at least six types of hyaluronidase-like enzymes, several of which are tumor suppressors. In most parts of the body, HA has a half-life of hours to a few days. Whereas synthesis occurs locally in the tissue, degradation takes place in various locations. Approximately 30% of the HA is turned over locally, while the other 70% is by lymphatic drainage. About 90% of that 70% is eliminated within the lymphatic nodes [35].

Hyaluronidases enhance connective tissue permeability and decrease bodily fluid viscosity. Hyaluronidases are recognized as the “spreading factor” due to the fact that they allow germs and poisons to get through infection into the skin and tissues. Most tissues exhibit expression of hyaluronidases. There are six types of hyaluronidases in humans: HYAL-1, HYAL-2, HYAL-3, HYAL-4, PH-20/SPAM1, and PHYAL134. The majority of the hyaluronidase family components are engaged in tumor-related action [36].

HA degradation products, oligosaccharides, and HA with a very low molecular weight display pro-angiogenic properties and promote inflammation [37]. Additionally, recent studies have shown that HA fragments, rather than the high molecular weight native molecule, can induce inflammatory responses in macrophages and dendritic cells in tissue injury and skin grafting [38,39]. Furthermore, there are also non-enzymatic reactions that allow HA to be degraded, including acid and base hydrolysis and degradation by oxidants [40]. Abnormalities in the synthesis, degradation, and metabolism of HA bring about serious organ dysfunctions and life-threatening diseases [41].

## 4. The Opposite Effect Related to the Molecular Weight of HA

Rapid HA turnover occurs in the presence of various forms of HA in both normal and pathological circumstances, each of which is distinguished by polymer length and, thus, molecular weight. To properly explain the significance of HA size in homeostasis and cancer, it is necessary to focus on the different molecular weight forms of HMW-HA, (Mw > 5 × 105), medium molecular weight HA (MMW-HA, Mw 2 × 104–105), LMW-HA, Mw 400–4000), and oligomeric HA (oligo-HA, Mw 6 × 103–2 × 104). The molecular weight of HA demonstrates a polydisperse distribution in human tissues in numerous circumstances, including cancer [12,42]. According to certain research, the numerous biological functions of the GAGs are significantly correlated with the size of the HA [11].

### 4.1. HMW-HA

In homeostasis, HA is present in its HMW form in practically all human tissues [8]. Endogenously formed HMW-HA-rich matrices are involved in the formation of several tissues, including the brain [43], hematological system [44], and heart [34]. In the human body, HMW-HA’s protective functions are observed not only in inflammation, embryogenesis, and wound healing, but also in cancer [28,45]. In a lipopolysaccharide-induced lung damage model, intraperitoneal therapy with HMW-HA effectively blocked monocyte and neutrophil infiltration [46]. HMW-HA has shown to suppress the migration and regrowth of cancer cells [47,48], as well as the production of anti-inflammatory mediators in several tumor models [49].

**Figure 2:**
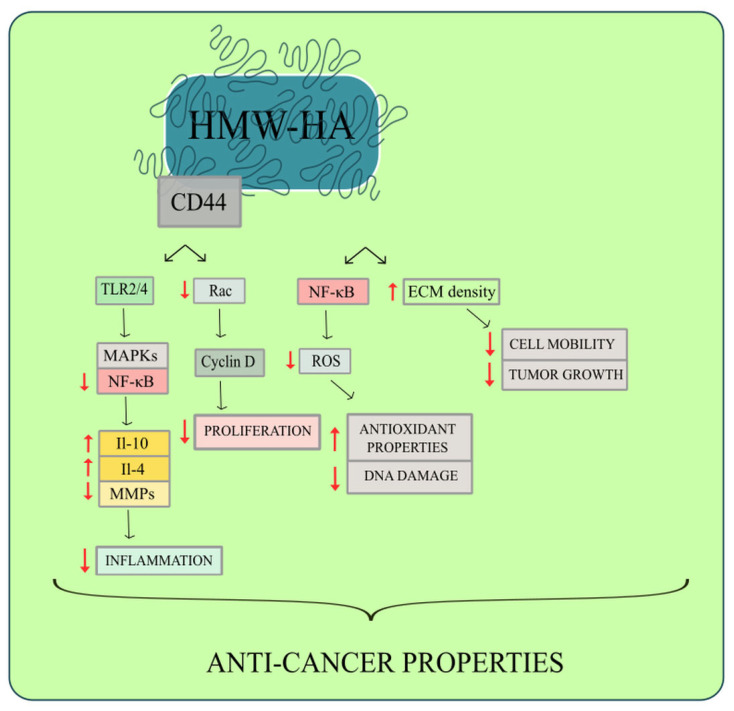
HMW-HA shows anticancer properties due to triggering a signaling cascade via the CD44 receptor. HMW HA binds to TLRs 2 and 4 and then inhibits MAPK and NF-κB pathways, exhibiting anti-inflammatory effects by the production of IL-4 IL-10. Additionally, this signaling pathway diminishes MMP activation. Moreover, Cyclin D1 is inhibited by Rac-dependent signaling when HMW-HA binds to CD44. As a result, cell cycle progression and cell proliferation are suppressed. HMW-HA also increases ECM density, which prevents cell mobility and tumor growth. The ability of HMW-HA to reduce ROS activity and DNA damage is caused by NF-B regulation.

In terms of antiangiogenic actions, HMW-HA has been shown to impede vascular smooth muscle cell growth by preserving the cells in the G1 phase [50]. HMW-HA reinforces cell-cell connections and lessens ECM permeability, which prevents an invasion. By generating a thicker ECM, HMW-HA might help prevent metastasis (Figure 2). HMW-HA treatment improved the monolayer integrity of cancer lymphatic endothelial cells, inhibiting cancer cell expansion [51]. Moreover, HMW-HA binding to CD44 specifically inhibits Rac GTP-loading and Rac-dependent signaling to the cyclin D1. As a result, cell cycle progression and cell proliferation are suppressed (Figure 2) [52]. HMW-HA has shown to suppress pro-inflammatory effects by reducing cyclooxygenase-2 (COX2) and matrix metalloproteinase (MMP) production and inhibiting MAPKs and NF-B activation [53].

Thanks to anticancer properties demonstrated after exogenous administration, HMW-HA is considered an appealing substance to enhance both adjuvant and neoadjuvant chemotherapy [49]. The prior analysis also showed that at physiological concentrations, HMW-HA defends human cells from stress more effectively than shorter HMW-HA [54,55]. HMW-HA also functions as an antioxidant, minimizing the damage caused by reactive oxygen species (ROS). It has been observed that HA can defend human skin fibroblasts from the carcinogenic effects of oxidative stress. NF-κB and caspases regulation contributes to HA’s ability to lessen oxidative damage in fibroblast cultures. The cell damage caused by FeSO(_4_) and ascorbate was diminished in fibroblasts after treatment with HA. Additionally, HMW-HA was found to have protective effects against mtDNA damage, which builds up after exposure to pro-inflammatory cytokines [56].

In recent years, studies on naked mole-rats (NMR, *Heterocephalus glaber*) have shown the existence of HA molecules 5 times larger than human HMW-HA. NMR is a long-lived mammal that has never developed a malignant tumor. The molecular weight and distribution of HA in NMR exhibit unusual characteristics. NMRs have higher levels of HA in their kidneys, brains, hearts, and skin than other species. The NMR produces vHMW-HA. NMRs evolved a larger quantity of HA in their skin to give the skin the flexibility required for survival in underground tunnels. Fibroblasts create a unique vHMW-HA that generates a tight layer surrounding the cells. This layer is thought to impede not only carcinogenicity but also the spread of tumor cells [12]. Reduced activity of HA-degrading enzymes and a specific sequence of HAS2 have been proved to cause extensive accumulation of vHMW-HA in NMR tissues [57,58].

Stimulation of intracellular processes and pathways by vHMW-HA accumulating in the ECM is essential for early contact inhibition (ECI) and suppression of oncogenic signals [59]. ECI is responsible for the inhibition of proliferation when cells interact with each other or with the ECM at significantly decreased densities [60]. The interaction of vHMW-HA with the CD44–NF2 pathway promotes contact inhibition, regulates ECI, and might trigger cell arrest [12]. The CD44 receptor interacts with neurofibromin 2 on the cytoplasmic surface. In NMR cells cultured with HAase, the phosphorylated, growth-promoting form of neurofibromin 2 was visible, but in cells cultured without HAase, the unphosphorylated, growth-inhibitory form of neurofibromin 2 predominated. In reaction to HA, neurofibromin 2 becomes hypophosphorylated at high cell density and inhibits cell proliferation. The particular interaction of Merlin with CD44’s cytoplasmic tail is necessary for its growth-inhibitory function. Neurofibromin 2 is phosphorylated, growth-permissive, and occurs in combination with CD44 at low cell densities. These findings suggest that cell growth arrest and proliferation are formed by neurofibromin 2 and CD44 [61]. ECI is one of the factors responsible for the cancer-resistance feature discovered in NMR. vHMWM-HA has been shown to induce ECI, which prevents hyperplasia and stops the cell cycle at low cell densities, protecting against cancer [12]. Furthermore, in response to vHMW-HA activation, NMRs create a unique tumor suppressor molecule, which encodes mammalian tumor suppressors: the cyclin-dependent kinase inhibitors p15, p16, as well as p19, a repressor of the MDM2 oncogene. It plays an important role in stopping the cell cycle during tumor development [62].

The NMR possesses another distinguishing feature in terms of TP53 and RB1 downregulation. Mutations of TP53 or RB1 are associated with cancerogenesis and aggressive cancer phenotypes [63]. In NMR cells, inactivating just one of these cancer inhibitors triggers apoptosis [64]. In contrast, the inactivation of either RB1 or TP53 in mice or human cells led to faster proliferation [65]. Previous studies indicated a hypothesis in which vHMW-HA blocks protein-protein interactions and CD44 activation, leading to differential expression of p53 controllers and targets [54]. This pathway is in charge of cell cycle arrest and cell death, essential actions in effective cancer therapy [66]. When Tp53 and Rb1 are inactivated, and vHMW-HA is suppressed in NMR cells, either through gene silencing or overexpression of an HA-degrading enzyme, the cells are more likely to develop malignancies [67]. The mechanisms underlying cancer resistance in NMR species are critical as they can be used to develop potential anticancer drugs.

### 4.2. Oligo-HA or LMW-HA

In comparison to HMW-HA, these shorter bioactive HA fragments can interact with cancer cells and alter their activity differently [68,69]. While HMW-HA is proved to maintain tissue homeostasis, HA breakdown products LMW and oligo-HA, generated by hyaluronidase (HYAL) or ROS, are often associated with enhanced invasion of cancer cells and tumor growth [9,70]. There is scientific proof for oligo-HA’s pro-inflammatory effects on tumor development and metastasis. Oligo-HA has been indicated to increase the proliferation of papillary thyroid carcinoma cells in vivo via a toll-like receptor (TLR) 4-dependent signaling pathway [71].

The quantity of oligo-HA in the tumor interstitial fluid of colorectal tumors may be connected with lymphatic invasion and lymph node metastases [72]. Additionally, oligo-HA contributes to increased angiogenesis. The angiogenic characteristics of HA fragments are mediated by either directly stimulating endothelial cell differentiation or raising the levels of angiogenic growth factors. Rapidly aggressive bladder tumors, for example, may release angiogenic HA fragments with 10–15 disaccharide units, which may also enhance endothelial cell proliferation, adhesion, and capillary development [73,74].

Oligo-HA also boosts the first stages of metastasis. Previous research has established that oligo-HA destroyed tight junctions in the cancer lymphatic endothelial cell monolayer and accelerated cancer lymphatic metastasis by decreasing cellular integrity [51].

Both tumor cells and tumor-associated stromal cells, such as fibroblasts and macrophages, may generate angiogenic factors to influence endothelial cell migration and differentiation as a consequence of oligo-HA activity [75]. Oligo-HA-mediated angiogenesis is an example of how tumors might use normal physiological capabilities that were previously assigned to healing processes. As a result, by altering biological pathways, distinct tumor cells can improve adhesion, angiogenesis, and invasion with the aid of oligo-HA [70]. The gradual weight loss of HA particles might diminish ECM density and more quickly facilitate cancer cell invasion into neighboring tissues. Metastasis and multidrug resistance (MDR) are raised as a result of oligo-HA presence. According to a recent study, oligo-HA weakens cellular integrity, damages tight junctions in the cancer lymphatic endothelial cell monolayer, and increases cancer lymphatic metastasis [72,76]. Additionally, activation of the receptor tyrosine kinase c-Met, commonly known as the hepatocyte growth factor (HGF), is enhanced by oligo-HA, leading to increased cell proliferation, differentiation, and penetration [77].

LMW-HA has been shown to increase cancer cell adhesion, proliferation, and MMP secretion for matrix remodeling. Additionally, pro-inflammatory and pro-angiogenic properties of LMW-HA have been noted, which may support the invasion of cancer cells [10]. Due to its correlation with metastasis in breast cancer, LMW-HA may be significant as a diagnostic marker in cancer [78].

In contrast to the tumor-promoting effect, small oligo-HAs (3–9 disaccharide units) are utilized in effective anti-tumor therapy. Synthesized small oligo-HAs can eliminate several types of tumor cells and stimulate apoptosis without affecting healthy tissue. In vivo melanoma development might be strongly suppressed by injecting HA oligomers to stimulate motility, invasion, and metastatic behavior. Researchers have shown that small HA oligomers (6–10 disaccharides) can overturn cancer cell adherence, migration, and invasion in some ovarian cell lines [79].

Oligo-HA has demonstrated other preventive properties in cancer, for instance, by interfering with the endogenous interaction of HMW-HA with CD44. Oligo-HA causes inhibition of tumor growth in a highly metastatic breast cancer cell line [69]. There is a discrepancy in the scientific literature regarding the role of oligo-HA in the neoplastic environment connected with stimulation and prevention of tumor growth. This may be due to the fact that tumors derive from a wide range of cells and develop as a consequence of various mutations.

## 5. Receptors of HA and Their Role in Cancer Biology

Based on numerous studies, it is known that different molecular weights of HA lead to different cell signals. There are two major HA receptors, known as hyaladherins: cluster differentiation (CD44) and the HA-mediated mobility receptor (RHAMM) [80].

CD44 belongs to the family of transmembrane glycoproteins, which contains diverse isoforms. CD44 receptors appear on fibroblasts, endothelial cells, leukocytes, keratinocytes, epithelial cells, hematopoietic cells, and various neoplastic cells. CD44 plays a significant role in cell adhesion, migration, and tissue formation. The adhesive and spreading activities of lymphocytes are also associated with CD44. The process of interaction of extracellular vesicles occurs through CD44 [81]. This receptor is crucial in physiological processes such as hematopoiesis and limb development [82]. Moreover, previous studies have confirmed that in human tumors the level of CD44 expression is increased [83]. The CD44 receptor occurs in numerous isoforms. The standard CD44 isoform is CD44s and appears in healthy tissues. In turn, CD44v is expressed during carcinogenesis. CD44 binds not only with HA, but also with different ligands such as collagen, fibronectin, and laminin due to exposure to post-translational modifications [84,85].

The level of CD44v expression is closely related to tumor progression. Additionally, it was identified that CD44 in cancer stem cells can renew itself [86,87] and facilitate the mobility of stem cells [88]. Notably, macrophages are characterized by the expression of CD44 receptors. Due to this fact, HA-based nanoparticles have been developed for targeted gene delivery systems to tumor-associated macrophages that convert the anti-inflammatory M2 type of macrophages to the anti-tumor M1 type and switch the immunosuppressive state in the tumor [89].

CD44 isoforms regulate cell function through receptors that are important for tumorigenesis. These receptors include receptor tyrosine kinases (RTKs) such as c-Met, vascular endothelial growth factor receptor-2 (VEGFR-2), platelet-derived growth factor receptor (PDGFR), and epidermal growth factor receptor (EGFR). They play a significant role in augmenting or suppressing several signaling pathways such as Wnt and Fas. These pathways have a substantial impact on the inhibition of the mechanisms related to the apoptosis process in cancer cells and development of drug resistance [82,90]. The linkage between HA and CD44 leads to tumor progression and cancer metastasis [83]. The interaction between HA and CD44 has been shown to have a relevant effect on the binding of cytokines, chemokines, and growth factors to the respective receptors. Thus, this interaction contributed to cell differentiation, migration, proliferation, and angiogenesis, leading to an increased propensity of cancer cells to metastasize [61,91].

Another receptor that binds to HA is RHAMM. It also plays a role in cell proliferation and migration. It is characterized by a low level of expression in normal tissues [92]. RHAMM shows increased expression in cancer cells, which might also lead to metastasis [93]. RHAMM is a predominantly hydrophilic helical protein that contributes to enhanced cell motility. It interacts with HA through positively charged amino acid clusters in the carboxyl terminus [82].

Due to its ability to interact with the cytoskeleton as well as with signaling molecules or diverse kinases, RHAMM is a key player in regulating cell motility and ECM restructuring. It is especially involved in the processes of tissue injury and wound healing. Besides, RHAMM has been found to regulate mitosis as well as the proliferation of fibroblasts and be expressed in a variety of cell types, including endothelial but also tumor cells [94,95,96]. Because higher RHAMM levels are correlated with a worse outcome in human malignancies, it seems that cancer cells use RHAMM’s injury-healing capabilities to support their longevity and development [97]. RHAMM protein and its mRNA expression are elevated in the majority of cancers, and these raised levels are linked with severe malignancies. According to the survey, hyperexpression of RHAMM mRNA is widespread in breast cancer, and this upregulation is also frequently correlated with poor clinical prognosis [98]. RHAMM expression is associated with diminished survival. These findings suggest that RHAMM might pose a prognostic factor in neoplastic diseases and become a potential therapeutic target in future treatments [99].

## 6. Role of HA in Cancer Progression

Recognizing the function of HA could lead us to a new approach to studying cancer control pathways. HA occurs in the environment of cancer cells and is a key participant in many cancer-related mechanisms. Many studies show that HA influences many features of cancer, such as development, invasiveness, and treatment resistance. The cancer-related effect of HA is contradictory and appears to be highly dependent on the type of tumor [24,49].

### 6.1. Expression of HAS

Increased *HAS2* activity may contribute to tumor-promoting effects by encouraging inflammation [100]. Variations in HAS expression, accompanied by unregulated HA production, result in HA buildup and HA-induced inflammation, which may also escalate cancerogenesis [49]. Additionally, elevated HA production signals are associated with tumor growth in a variety of malignancies by increasing cell motility and growth [27]. According to research, reduced expression of *HAS1*, *HAS2* is observed in highly metastatic melanoma and associated with an adverse outcome [101]. Contrarily, HAS1 expression is elevated in patients with high-grade bladder tumors, and it may be linked to recurrence, clinical outcome, and disease progression [102]. *HAS2* overexpression in prostate cells enhanced angiogenesis but had no effect on the size of subcutaneous tumors [103]. *HAS2* promotes the invasion of breast cancer cells by decreasing tissue metalloproteinase inhibitor 1 (TIMP-1) [104]. Conversely, inhibition of *HAS2* leads to reduced tumor cell migration, diminished tumor cell proliferation, and improved chemotherapy sensitivity in breast cancer and cells [105,106].

It has been shown that the levels of enzymes taking part in the synthesis and degradation of HA are higher in tumors and may work cooperatively in malignant progression. Elevated HA production by cancer cells boosts their proliferation only if the cells also express hyaluronidases. Such particular interplay between HASs and HYALs has been proved in prostate cancer cell lines [103]. Moreover, co-expression of the *HAS2* and *HYAL1* genes resulted in increased cell mobility and metastasis in vivo [107]. The quantity of HA buildup surrounding cancer cells and in the neighboring stroma closely corresponds with malignancy aggressiveness by promoting cancer development processes [108]. By increasing the expression and activity of MMP-7, the inhibition of *HAS2* and/or HAS3 reduced colon cancer cells’ ability to spread [109]. Furthermore, *HAS1* and *HAS3* knockdown reduce cancer progression and metastasis in breast and osteosarcoma models [110]. *HAS3* overexpression promotes tumor cell proliferation and extracellular matrix deposition in prostate cancer cells [111]. HAS2 has been shown to control tumor development and cell aggressiveness in many studies [112]. Breast cancer cell lines with an aggressive phenotype (MDA-MB-231 and HS578T) show a high level of HAS2 mRNA [97]. Furthermore, *HAS2* expression is significantly higher in bone metastases than in parental MDA-MB-231 cells, demonstrating that *HAS2* expression plays an important role in cell migration and invasion [113]. HAS2 is one of the key enzymes activated by cancer-associated fibroblasts; therefore, cancer cells may stimulate stromal cells to generate HA [114]. On the other hand, a recent study revealed that overexpressing *HAS2* prevented the growth of tumors and indicated the significance of maintaining a balance between the production of HA and its breakdown by hyaluronidase. A study found that overexpressing *HAS2* in the fibroblast cell line HR3Y1 suppressed the growth of peritoneal and subcutaneous tumors. According to the levels of HA synthesis, the expression of the *HAS2* led to aggressive cancer progression, but at high levels, the tumor growth was markedly reduced [100]. The effects of up- and downregulation of *HAS 1–3* in various types of malignancies have been summarised in Table 1.

### 6.2. The Role of Hyaluronidases in Cancerogenesis

Hyaluronidase is prominently expressed in many types of malignancies [36]. Moreover, a high level of hyaluronidase is also linked to tumor aggressiveness [115]. Products of hyaluronidase activity enhance angiogenesis; therefore, this enzyme may be an indicator of cancer development and metastasis [116]. HYAL-1 and HYAL-2 functions are crucial in carcinogenesis; however, there is a lot of inconsistent information about their specific role in tumor growth. According to data from several studies, hyaluronidases appear to be both tumor accelerators and growth arresters [117]. In research conducted in vitro and in vivo, tumor cell growth and mobility were increased by forced HYAL-1 expression, while its knockdown reduced the proliferation of breast cancer cell lines [47]. These aberrations may be the result of variations in tumor strategy at various phases of tumor origin, development, and metastasis. HYAL-1 acts as a tumor enhancer at naturally occurring levels. Tumor progression can be inhibited by the activation of apoptosis by HYAL-1 levels exceeding 100 mU/10^6^ cells, which are not normally produced by cancer cells. Reducing HYAL-1 expression in bladder and prostate cancer cells inhibits tumor development and decreases their capacity to metastasize by triggering cell cycle arrest in the G2/M phase [116]. Overexpression of HYAL-1 accelerates the development of a mammary tumor and leads to extensive tumor angiogenesis [118]. In xenograft models of breast, bladder, and colon cancer, HYAL-1 has proved to promote tumor mobility and penetration [119,120,121]. Multiple data indicate that HYAL-1 expression is associated with tumor progression and poor clinical outcomes. In one study, reduced HYAL-1 activity promoted carcinogenesis in tobacco-related head and neck cancers [122]. This suggests that HYAL-1 may potentially act as an oncogene [123]. Nonetheless, several findings question the idea of HYALs acting as cancer promoters. For example, overexpression of HYAL-1 inhibited carcinogenesis in rat colon cancer cells [121]. Whereas upregulation of HYAL-2 inhibited tumor development in experimental lung metastases in mice [124].

HYAL-1 and HYAL-2 are upregulated in prostate cancer, bladder cancer, and melanoma. In contrast, under-expression of HYAL-1 and HYAL-2 may contribute to poorer prognosis in pancreatic [125], ovarian [126], and endometrial malignancies [127], suggesting that the detrimental effects of hyaluronidases in cancer might vary depending on the type of cancer [128].

Previous research revealed that HYAL-2 acts as a TGF-1 receptor by activating tumor suppressors WW domain-containing oxidoreductase (WWOX) and Smad4. Their overexpression indicates the presence of the apoptotic process [129,130]. Inconsistent results suggest that HYAL-1 and HYAL-2 might both stimulate and prevent tumor development and progression. However, modulation of HYAL-1 and HYAL-2 activity may be part of a complex regulatory mechanism involving synthesis and degradation pathways [70,115]. HYAL-1 might be a diagnostic indicator of epithelial ovarian malignancies [131], as well as bladder cancer [132], through elevated levels in the serum.

Anticancer treatments have already included hyaluronidases. Nevertheless, it has been shown that administration of hyaluronidase to a tumor that was previously drug-resistant may develop responsiveness. Hyaluronidases operate in these schemes by increasing the absorption of anticancer medications [133]. Recombinant human hyaluronidase is currently approved and applied to facilitate the absorption of several injectable medicines. In patients with the most lethal forms of cancer, hyaluronidase may enhance therapeutic efficacy [134].

The effects of up- and downregulation of *HYAL 1* and *2* in various types of malignancies have been summarised in Table 2.

### 6.3. The Influence of HA on the Immune System

**Figure 3:**
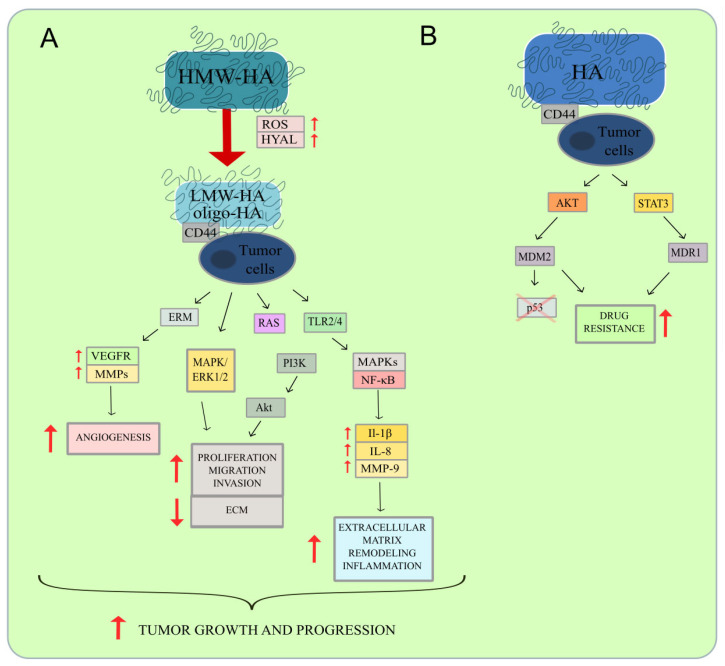
(**A**) Hyaluronidases and reactive oxygen species decompose HMW-HA into LMW-HA or oligo-HA. In the cancer environment, these molecules bind to CD44 on tumor cells and trigger various signaling pathways. For example, CD44 binds to ERM proteins, which interact with VEGFR. Thus, levels of MMPs are increased, contributing to angiogenesis. Furthermore, LMW-HA and oligo-HA interact with CD44 to trigger MAPK/ ERK1/2 activation. The stimulation of these pathways leads to increased cancer cell proliferation, migration, invasion, and ECM degradation. CD44 combined with LMW-HA and oligo-HA also promotes the RAS/ PI3K/Akt signaling pathway, which supports tumor growth and progression. Through interactions with CD44 and tumor cells, fragments of HA degradation activate MAPKs, the NF-κβ pathway, by modulating TLR2/4 downstream signaling. This signaling increases the expression of inflammatory cytokines, including IL-1β, IL-8, and MMP-9, to create a tumor-promoting environment by remodeling the ECM and inducing inflammation; (**B**) HA-CD44 signaling on tumor cells may enhance the phosphorylation of MDR1 by activating STAT3 or cause AKT/MDM2 stimulation, which blocks the p53 genomic surveillance response to mediate multidrug resistance.

Several studies have been conducted to investigate the effect of HA on the production of pro- and anti-inflammatory cytokines [135]. LMW-HA or oligo-HA bind to TLRs 2 and 4 and activate NF-κB MAPKs signaling, which leads to enhanced inflammatory responses. This mechanism was demonstrated in melanoma cells resulting in increased production of matrix metalloproteinase 9 (MMP-9) and the inflammatory cytokines IL-8 and IL-1β, which may promote tumor growth (Figure 3A) [136]. Oligo-HA-induced physical interaction between the primary HA receptor CD44 and TLR2, and TLR4 triggers pro-inflammatory cytokine and chemokine production in breast cancer cells [68,137].

HA fragments might alter neutrophil activity in the cancer surroundings. Cancer cell-derived HA particles trigger the initial activation and survival of cancer neutrophils, which in turn enhances malignant cell migration [138]. On the other hand, HMW-HA inhibits MAPK and NF-κB signaling, implying anti-inflammatory properties through the production of IL-4 and IL-13 [53]. An increased level of HMW-HA results in decreased inflammation and lower biomechanical stress [139]. Additionally, HMW-HA was proved to stimulate IL-10 production, thereby reducing inflammation in the tumor environment (Figure 2) [140].

### 6.4. Cancer Signaling Pathways

HA plays an important role in cancer signaling pathways leading to proliferation and metastasis. Pro-tumorigenic behavior mediated by HA includes cancer cell motility, invasion, adhesion, angiogenesis, or multidrug resistance [28]. Cancer cell development is not exclusively dependent on genetic changes, but also on interactions with the tumor microenvironment, which is constituted of stromal cells such as fibroblasts, cancer-associated fibroblasts (CAFs), and a range of immune cells that are located within the ECM. Fibroblasts’ ability to produce more HA has been shown to facilitate CAF activation [141].

The anchorage-independent growth of cancer cells is a significant component of cancer form, particularly in terms of spreading ability. This process appears to be an important part of tumor biology as it is related to tumor aggressiveness [142]. In human cancer, the phosphoinositide 3-kinase (PI3K) pathway is commonly upregulated. Genetic alterations in these signaling cascades play a critical role in cancerogenesis [143]. Anchorage-independent cancer cell growth is enhanced by HA and mediated by subsequent activation of the PI3K/Akt survival pathway. LMW-HA stimulates the activation of the RAS/PI3K/Akt pathway, which promotes the development and progression of cancer, as well as the epithelial-mesenchymal transition responsible for metastasis (Figure 3A). However, this process can be suppressed by blockers of HA oligomers [144,145].

Various signaling pathways are indirectly stimulated by the interaction between HA and receptor CD44, which promotes cancer progression as well as the transcription of pro-oncogenic genes. Studies performed by Zhao et al. have shown that HA-CD44 stimulates the proliferation of lung cancer cells by activating the pro-oncogenic MAPK pathway. Additionally, blockade of the CD44 gene resulted in inhibition of the Kras-mediated development of lung adenocarcinoma in mice [146]. It has been shown that stimulation of c-Jun is caused by the HA-CD44 connection. Thus, apoptosis is suppressed by increasing the levels of the anti-apoptotic protein Bcl-2 [147].

The MAPK/ERK pathway plays a crucial role in the survival and development of tumor cells. ERK cascades are responsible for basic cellular processes, including cell proliferation and differentiation [148]. Protein kinase C (PKC) and finally mitogen-activated protein (MAP) kinase (ERK-1 and ERK-2) are both activated when oligo-HA binds to the CD44 receptor. This cascade signaling leads to the induction of proliferation (Figure 3A) [149]. Furthermore, LMW-HA binding to CD44 promotes cell cycle progression by selectively activating ERK and ERK-dependent cyclin D1 gene expression [150]. RHAMM and CD44 were shown to interact in an HA-dependent autocrine system to regulate signaling via ERK1,2, resulting in increased motility of invasive breast cancer cells [151].

The activation of the CD44 receptor by HA appears to inhibit apoptotic cell death. Research conducted by Lakshman et al. on a CD44 knockout mouse model of colon cancer revealed high levels of apoptosis regulated by the mitochondrial pathway [152]. In contrast, cancer cells that have high rates of CD44 are more resistant to apoptosis [153]. The induction of apoptosis was accomplished by utilizing anti-CD44 antibodies [154]. This might offer a novel treatment intervention strategy based on accelerated tumor cell death.

### 6.5. Reactive Oxygen Species

ROS are produced in locations of tissue damage, inflammation, and the tumor microenvironment. In vivo, ROS causes the formation of HA fragments, which can further aggravate inflammation [155]. Tumor tissues exhibit enhanced formation of ROS as a consequence of higher metabolic activity, increased activity of NADPH oxidase (NOXs), or mitochondrial malfunction. Studies proved that the breakdown of HA in the tumor environment, caused by increased HYAL expression and higher ROS accumulation, leads to the invasion of neoplastic cells [115,156]. The chemical change of HA caused by ROS may have a negative impact on the formation, structure, and repair of the ECM [157]. The HA-rich ECM is created by the action of ROS. It contributes to the recruitment of mesenchymal stem cells, which are the source of CAFs, supporting tumorigenesis and tumor angiogenesis [75].

Data from several studies have identified that HMW-HA also functions as an antioxidant, decreasing the destruction caused by ROS. HA pre-treatment of chondrocytes resulted in a reduction in mitochondrial DNA damage and an increase in mitochondrial DNA repair capability. HMW-HA treatment has been shown to increase cell viability by reducing the number of apoptotic cells. The cleavage of procaspase 9 and the release of cytochrome c from chondrocyte mitochondria have been inhibited by HMW-HA. The therapeutic effects of HA are linked to improved mitochondrial activity and higher chondrocyte survival in the presence of oxidative stress [158]. A study showed that the creation of native HA guards against DNA damage caused by UVB or gamma radiation [159]. Native HA in the skin has also been associated with a lower incidence of skin cancer and metastases. The HA matrix may be an effective barrier to stop the spread of melanoma cancer cells due to its ability to scavenge harmful ROS or prevent their production [160].

Studies have found that HA and chondroitin-4-sulphate (C4S) may lessen cell damage under oxidative stress by suppressing NF-kB and apoptosis activation. Furthermore, the chelation of transition metal ions is responsible for this antioxidant activity [56]. The source of HA’s defensive function might involve the immobilization of iron ions, consequently blocking the Fenton’s reaction, which forms secondary oxidative species, or direct scavenging of primary and secondary ROS as an antioxidant [161]. Additionally, HA might prevent ROS generation by reducing the proportion of apoptotic cells and suppressing caspase 3 and 7 [162].

It has been demonstrated that HMW-HA exhibits antioxidant and anti-apoptotic properties through TLR-dependent NF-κB activation (Figure 2). HMW-HA and CD44 binding has been reported to inhibit glutathione (GSH) production, induce apoptosis resistance, and contribute to ROS protection. Several proposed pathways for HMW-HA protection involve regulatory T-cell activation, adaptive immunity suppression, and TLR4-signaling negative regulation [163].

### 6.6. Chemoresistance

Various studies have elucidated the function of HA in clinical outcomes in neoplastic diseases. The HA-CD44 signal in cancer cells may promote multi-drug resistance. CD44 causes increased activation of AKT to promote phosphorylation and nuclear translocation of MDM2 proto-oncogene, which blocks the p53 genomic surveillance response (Figure 3B) [164]. MDM2 activation has been linked to resistance to conventional chemotherapy [165]. Research performed by Bourguignon et al. showed that Nanog, a cytoskeletal protein, and ankyrin are activated in breast and ovarian cancer cells by HA-CD44 binding. While ankyrin binds directly to MDR1/P-gp, Nanog forms a complex with the activator of transcription protein 3 (STAT3). It stimulates signal transmission and regulates the production of the multidrug transporter MDR1 (P-glycoprotein). Both mechanisms ultimately result in tumor development and chemoresistance and contribute to the efflux of chemotherapeutic drugs (Figure 3B). The overexpression of pumps that remove medicinal substances from the cell is one of the processes activated by these pathways [166].

Additionally, CD44 interactions with HA stimulate a variety of signaling pathways that contribute to the activation of pro-survival signaling, including stimulation of MDR1 expression, leading to drug resistance. CD44 has been demonstrated to promote drug resistance in osteosarcoma cells by increasing MDR1 levels and affecting apoptosis-related genes. In contrast to chemotherapy-responsive individuals, MDR1 expression levels in non-responding patients were noticeably higher [167].

In cancer, HA can control the expression of several ABC transporters. The efflux of cancer drugs and activation of pro-survival and anti-apoptotic pathways are caused by the upregulation of ATP-binding cassette ABC transporters. The PI3K pathway was activated by HAS2 overexpression in breast cancer, enhancing the expression of ABCB1/MDR1 and thus contributing to treatment resistance [168].

Certain cancer cells might upregulate HA during treatment by chemotherapeutic agents, resulting in a tumor-protective effect [169]. Another theory is that high levels of HA might inhibit chemotherapeutic drugs from contacting cancer cells [49]. Additionally, HA has been shown to promote the formation of cancer stem cell (CSC) populations, which are hypothesized to be responsible for the initiation of tumors and therapy resistance [170].

### 6.7. Angiogenesis and Tumor Cell Metastasis

Current research has indicated that the synthesis of HA by epithelial or stromal cells may be valid for the proliferation and vascularization of carcinomas. Additionally, concurrent excessive HA production and degradation may raise the metastatic potential [171]. LMW-HA and oligo-HA formations bind inflammatory cells into tissue, and tumor-associated macrophages generate angiogenic and lymphangiogenic growth factors [168]. Angiogenesis is induced in vivo by HA breakdown products of a certain size (3–25 disaccharide units) [172]. Additionally, oligo-HA fragments of 3–10 disaccharides have been found to stimulate EC migration, proliferation, and tube formation [173]. HA-CD44 may also be associated with the formation of blood arteries. Thus, cancer cells are transported to distant areas of the body, which supports tumor growth. [174]. Moreover, 35-kDa HA increases vimentin expression and decreases E-cadherin expression, thereby enhancing cellular migration and invasion [175].

The HA–CD44 interaction results in the upregulation of MMPs, which decompose the ECM promoting angiogenesis and metastasis [176]. The HA oligo-saccharides have been demonstrated to encourage angiogenic capillary sprouts that penetrate the fibrin/fibronectin-rich clot site and build a microvascular network that supplies the expanding tissue [177,178]. Cancer cell spread is promoted by HA breakdown products, especially HA oligosaccharides. HA fragments during interaction with CD44 might enroll ERM proteins, which can interact with VEGFR, contributing to angiogenesis by promoting vessel formation and tumor cell migration [179]. It leads to MMP-mediated degradation and remodeling of ECM (Figure 3A) [180]. The HA-dependent CD44 receptor has been shown to proteolytically activate MMP-9 on the cell surface leading to the promotion of tumor invasion and angiogenesis. Moreover, MMP-9, like MMP-2, has been shown to proteolytically cleave TGF-beta. These findings imply that the coordinated action of CD44, MMP-9, and TGF-beta is responsible for the tissue remodeling process that cancer cells might employ to promote tumor development and migration [181]. Additionally, the interaction of HA with the CD44 receptor has been shown to stimulate the RhoA protein and increase the level of intracellular calcium, which may contribute to the facilitation of metastasis. RhoA protein exhibits GTPase function, and its role is to participate in intracellular signaling [182]. This protein leads to molecular changes that allow the migration of cancer cells and is crucial for the development of tumors [183].

## 7. Hyaluronic Acid—Therapeutic Target and Chemotherapy Enhancer

Even though numerous therapies for various forms of cancer are now accessible, most of them have severe side effects and are harmful to healthy tissues. One of the primary aims of drug development is to design a treatment or preventative method that is both impactful and non-toxic. Chemotherapy is now the most common treatment method for a significant number of malignancies. Chemotherapeutics are generally cytotoxic but target high-proliferating cells, which have the ability to target the skipping cell cycle checkpoints. The key aim of cancer therapy techniques is to eradicate cancer cells and simultaneously improve patients’ lifespans. HA is a popular and common substance used for medical applications, including tissue regeneration, anti-aging, and anti-inflammatory properties [23,184]. Additionally, HA shows good biocompatibility, enzymatic degradation, and easy modification. Therefore, it is used successfully as a nanocarrier for drug delivery in cancer chemotherapy [185]. In numerous studies, the use of HA-based nanocarriers as a drug delivery method has been linked to decreasing tumor growth, enhancing the patient’s quality of life, and minimizing drug toxicity [186]. Also, due to their properties, HA-based nanocarrier drugs are a promising target for drug delivery in oncological diseases [187].

Recent data suggest that HA-based drug nanocarriers have a significant impact on improving drug distribution to cancer cells. Moreover, it was found that the use of HA-coated lipid nanoparticles as a biocompatible drug carrier has great potential for targeted drug delivery. Additionally, it does not affect other tissues and reduces the side effects of cancer therapy [188]. Considering HA’s great affinity for the CD44 receptor on tumor cells, there has been much interest in using it as a desirable coating on the surface of therapeutic nanocarriers [189].

Receptors with a high binding affinity for HA are overexpressed in many tumor cells, whereas these receptors are underexpressed in healthy body cells. Because of these advantages, HA has been widely studied as a reinforcing agent for developing modern clinical cancer therapeutics in a variety of formulations. HA has been shown to augment drug delivery, thus increasing the efficacy of the anticancer drug, and notably suppressing tumor growth [190].

One of the methods used to target cancer cells is the interaction of the HA-CD44 receptor with nanoparticles. Cancer cells are considered to proliferate excessively and metastasize by HA-CD44 linkage. Interrupting HA-CD44 binding results in the deactivation of the pro-cancer pathways and minimization of the aggressiveness of cancer cells. For instance, the disruption of HA-CD44 binding by HA fragments results in cell death and inhibition of the PI3K/Akt cell survival pathway [69,191].

Furthermore, apoptosis in cancer cells may be triggered by anti-CD44 antibodies. Antibodies against highly expressed variations are also used to selectively deliver cytotoxic drugs to cancer cells. In early-phase clinical studies, anti-CD44v6 antibodies conjugated with the cytotoxic agent mertansine were utilized. Due to anti-CD44v6 treatment, the condition of the patients with head and neck, or breast tumors was found to improve [192]. Additionally, CD44 has been targeted in anticancer treatment, including the use of DNA vaccines, anti-CD44 mAbs, and nanoparticle-mediated administration of CD44 siRNA [193].

Using soluble hyaluronan-binding proteins, such as the soluble extracellular version of CD44 (sCD44) or the soluble form of RHAMM, is another method for preventing hyaluronan from interacting with its receptors. These proteins bind endogenous hyaluronan, lowering its ability to interact with membrane receptors. As a result, signal transmission is impaired, preventing many cancer cell lines from proliferating, invading, or metastasizing. Furthermore, it was proved to support the process of apoptosis [194].

HA has been applied in nanoparticle formulations for the targeted distribution of chemotherapeutic medicines and other anticancer chemicals to cancer cells via cell-surface HA receptors [195]. PEP1 (a 12-mer peptide that prevents HA from attaching to its receptors), hyaluronidase (which accelerates HA breakdown), and 4-Methylumbelliferone (4-MU) (an HA synthesis inhibitor) all diminish HA activity and are already used as an efficient oncological treatment [196]. As explained previously, HA accumulation as a result of synthase hyperactivation might lead to malignant transformation. Thus, inhibiting the enzymes might be a promising anticancer strategy. 4-MU is choleretic and an antispasmodic agent used in gastroenterology to treat motor abnormalities of the bile ducts. 4-MU has been extensively studied as a hyaluronan synthase blocker and presented as a possible cancer therapy option. 4-MU has been shown to decrease the proliferation and mobility of cancer cells and promote apoptosis [197]. It diminishes tumor microvessel density and the formation of distant metastases in vivo [198]. In a rodent model, 4-MU has been demonstrated to sensitize human pancreatic cancer cells to the oncological medication [199]. It has the benefit of being uncomplicated to administer orally and having minimal cytotoxic activity [198].

For almost 60 years, sulfated oligosaccharides of hyaluronan (sHA) have been required to decrease the enzymatic activity of hyaluronidases. Nevertheless, recent studies have shown that sHA suppresses the proliferation, mobility, and metastasis of several prostate cancer cell lines and promotes apoptosis. sHA are particularly efficient in suppressing tumor development in xenograft studies [200,201].

Chemically altered HAmolecules are effective carriers of anticancer drugs. Doxorubicin (DOX) and microcapsules have shown to combine effectively with acetyl derivatives of LMW-HA- Ac-HA. In vitro experiment has revealed that the microcapsules show a strong affinity for HeLa tumor cells and considerably increase the cytotoxicity of doxorubicin. In contrast, no cytotoxicity was seen in cells that did not produce hyaladherins. HAmicrocapsules that have been acetylated appear to be a potential strategy to boost the effectiveness and safety of commonly used anticancer chemotherapeutic agents [202].

DOX is one of the best-known anthracycline antibiotics used in cancer treatment. Although this drug is widely used in chemotherapy, it can cause dose-dependent toxicity. Doxorubicin linkage with HA was shown to possess excellent anticancer effectiveness and specific selectivity for CD44 receptors and otherwise remarkable bloodstream durability with minimal harmful impacts [203]. A Stable HA-DOX conjugation was obtained via amide linkage of DOX to HA using carbodiimide coupling chemistry with 1-ethyl-3-(3-dimethylaminopropyl) carbodiimide hydrochloride (EDC · HCl) and N-hydroxybenzotriazole (HOBt) as a catalyst. HA-DOX molecules were stable in the patient serum. Moreover, in vivo toxicity caused by the initial circulation of the drug in the bloodstream was significantly reduced. Accordingly, polymer-drug conjugates may be an effective therapeutic strategy for metastatic tumors. In previous research, HA-based nanoparticles containing DOX showed significantly fewer side effects compared to the free drug. HA cross-linked nanoparticles provide a great prospective treatment in cancer therapy owing to considerable biological compatibility [204].

Another example is increasing concentrations of H_2_O_2_ with simultaneous administration of HA, which has revealed that cancer radiosensitivity is augmented by use of H_2_O_2_  + HA. HA has been shown to also diminish H_2_O_2_ adverse effects. In 4T1 breast cancer cells, HA has been demonstrated to induce multi-phase cell cycle arrest, limit proliferation, and enhance apoptosis under hypoxic conditions. In turn, 4T1 cells’ propensity to migrate; and the levels of VEGF, MMP-2, and MMP-9 have all been substantially reduced. The antitumor effects of HA with H_2_O_2_ are strong and amplified, which is consistent with earlier observations on the usefulness of HA as a drug delivery agent [205].

On the other hand, RHAMM has been shown to stimulate either humoral or cellular immune responses. In phase I/II clinical trials, the potential of a peptide-based RHAMM vaccine was investigated. In a phase I clinical study, 10 patients suffering from AML, myelodysplastic syndrome, or multiple myeloma who possessed cancer cells expressing HLA-A2 and RHAMM were vaccinated with the RHAMM peptide R3. Subcutaneous delivery of this vaccine enhanced R3-specific CD8+ T cells, and three patients had an incomplete recovery, including a marked decrease in bone marrow blasts. The vaccine generated significant improvements in distinct T-cell subsets and immunological response cytokines (i.e., TGF-, IL-10, IL-2, and TNF) throughout the vaccination period [206].

The use of therapy with HA molecules seems to be promising. Studies have shown that the simultaneous administration of oligo-HA with anticancer drugs significantly increases the effectiveness of the therapy and may be able to tackle chemoresistance. The exogenous application of oligo-HA transformed chemoresistant tumor cells into drug-sensitive cells, suggesting that oligo-HA may provide a novel basis for the creation of anti-cancer medicines [207,208,209]. For instance, oligo-HA may make different tumor cell lines sensitive to chemotherapy, including lymphoma cells [210], ovarian carcinoma cells [169], and myeloid leukemia cells [211]. oligo-HA and LMW-HA are indicated to be effective in various forms of cancer since they can reduce cancer cell proliferation, motility, and invasion. In colon cancer cells, oligo-HA therapy inhibits the activity of cyclooxygenase-2. Therefore, HA production is reduced [212]. Additionally, oligo-HA stimulates caspase-3, which suppresses tumor development in vivo and promotes apoptosis in breast and colon cancer cells [213]. Treatment with HMW-HA improved cancer lymphatic endothelial cells’ monolayer integrity, inhibiting the spread of cancer cells. HMW-HA is considered an appealing drug to assist both adjuvant and neoadjuvant chemotherapy because of the anticancer effects demonstrated after exogenous administration [51].

HA fragments depending on defined molecular size can inhibit the proliferation, migration, and invasion of MDA-MB-231 breast cancer cells [214]. Additionally, HA might limit osteolytic activity, thus reducing bone metastasis growth [69].

The application of hyaluronidases to destroy HA in the cancer environment has also been investigated as a possible anticancer drug. Bacteriophage hyaluronidase was shown to efficiently suppress breast cancer cell proliferation, motility, and penetration [134]. The addition of hyaluronidase to anticancer medications has been studied several times, increasing the effectiveness of cancer therapy regimens [156]. This family of enzymes has not only been attributed to improving the bioavailability of anticancer medicines. Intravenous hyaluronidase treatment of mice implanted with human breast cancer cells resulted in a 50% reduction in tumor size within 4 days [215]. It is assumed that the breakdown of HA in cancer tissues may cause permanent alterations in their metabolism. There is scientific evidence that hyaluronidase therapy prevented lymphoma from migrating to the lymph nodes [216]. In clinical studies, hyaluronidase therapy has also exhibited promising results in pancreatic cancer [217]. Moreover, recombinant human hyaluronidase (rHUPH20) was found to enhance the drug delivery of trastuzumab-based targeted treatment in HER2+ breast cancer patients [218]. According to previous research, HA hydrolysis by pegvorhyaluronidase (PVHA) might alter the TME in a mouse model of breast cancer, enhancing the absorption of the anti-programmed death-ligand 1 (PD-L1) therapeutic antibody. PVHA therapy is correlated positively with an anti-PD-L1 antibody’s remarkable achievement in suppressing cancer progression [219].

Many research studies demonstrate that due to its physicochemical features, HA-based therapy can be utilized as a basis for targeted chemotherapy, gene therapy, immunotherapy, and combination therapy, with promising future biological applications in anticancer treatment.

## 8. Hyaluronic Acid as a Promising Biomarker

In 1939, Elvin Kabat established that Rous sarcoma tumor cells in chickens produce HA. In both animals and humans, the quantity of HA in malignant carcinoma tissues is higher in comparison with benign lesions and healthy tissues [75]. Cancer patients’ plasma also contains HA. There’s a correlation between increased HA production and secretion into the bloodstream and cancer’s invasiveness and inclination to spread [220]. Levels of HA might be a valuable diagnostic tool for cancers and a predictor of progressive disease. Many studies have indicated that HA has been linked to carcinogenesis, and high levels of HA have been linked to a variety of epithelial and connective tissue malignancies [221]. Furthermore, higher HA content has been found in bodily fluids such as the urine of patients with bladder carcinomas [222], serum of patients with breast cancer [223], saliva of patients with head and neck cancer [224], and interstitial fluid of colorectal tumors [225]. HA is more commonly concentrated in the stroma surrounding tumors than in the tumor parenchyma [226]. High stromal HA levels were reported in patients with breast [227,227] and ovarian carcinomas [228,229], as well as individuals with lung [230], brain [231], and prostate cancer [232]. However, an excessive HA deposition may be caused by cancer cells themselves [233]. As an example, the amount of HA in the parenchyma is correlated with lung [230], gastric [234], and colorectal malignancies [235]. As a result, a considerable number of studies revealed that in cancer patients, HA concentrations are often increased in tumors than in surrounding healthy tissues [108]. Consequently, increased HA deposition, which frequently corresponds with changes in HA polymer size, can be considered a key predictor of malignancy [236]. Plasma detection is widely characterized by its ability to offer relevant information at an appropriately early time. Scientists could evaluate the predictive relevance of HA in the plasma of oncological patients. There is no standard method for HA threshold values; therefore, their use in typical clinical practice is limited. Accordingly, the HA cut-off level should be determined in relation to the world population [13].

HA synthesis is essential for tissue regeneration. However, overexpression of the gene that is responsible for HA synthesis may contribute to cancer development and the unfavorable prognosis of many malignancies, especially breast cancer. Thus, HAS1 expression is increased in cell carcinomas and is a factor of poor prediction. It has been shown that HAS1 expression is related to poor mortality in multiple myeloma. Thus, these clinical data support the research observations on HAS1’s functional role in cancer development and differentiation [237].

Previous studies have assessed that high levels of HA are found in the environment of promptly growing tumors [134]. CD44 receptors are upregulated on the surface of multiple neoplastic cells, in particular breast cancer [238] and lung cancer [239]. As a result, CD44 can be used as a biomarker to target cancer. A study found that chemo- and radio-therapy, which are commonly used in cancer treatment, are less effective in the CD44 sub-population. Moreover, these cells show increased proliferation [240]. Accordingly, CD44 receptors might be posing as a relevant marker of treatment response and efficacy.

## 9. Conclusions

Despite extensive therapeutic strategies, cancer mortality is rapidly increasing worldwide. Also, the poor efficacy and severe side effects of a variety of treatment regimens in many cases remain an issue. HA, which was previously thought to exclusively have a structural purpose, now appears to play a part in the neoplastic process as well. It has been revealed that it participates in the adhesion, migration, invasion, and metastasis of cancer cells, including the process of angiogenesis. Together with receptor CD44, endogenous HA is also involved in different stages of malignant progression. HA expression is usually upregulated in the cancer microenvironment. The interactions between HA and the membrane receptors of cancer cells activate several pathways that promote tumor cell growth, thereby increasing metastatic spread. The involvement of the HA family of molecules in cancer development and progression, as well as their importance as biomarkers in practically all forms of malignancies, is being shown in a rising number of publications. The cancer-associated HA metabolism system is an appealing target for cancer therapy because it regulates major intracellular pathways, as well as mechanisms like epithelial-mesenchymal transition, which influence cell proliferation, invasive traits, and angiogenic ability. Also, the catabolic enzymes and breakdown compounds of HA have an elaborate impact on tumor growth. The molecular weight of HA determines its activities. HMW-HA has proved to inhibit mitogenic processes and shows anti-inflammatory effects, whereas LMW-HA has been shown to enhance proliferation and inflammation. Moreover, HA exhibits good biocompatibility, enzymatic degradation, and easy modification. Due to its properties, it is a promising target for drug delivery in oncological diseases. The application of HA nanosystems also has the potential to deliver chemotherapeutic medicines and other anticancer agents in a targeted and safe manner. According to the scientific literature, HA nanoparticles may increase the half-life of anticancer medicines and target delivery to cells overexpressing HA receptors. Therefore, many antitumor treatments may be more beneficial at lower dosages, resulting in fewer drug-related side effects. On the other hand, cancer-derived hyaluronidases have demonstrated the possibility to be a significant prognostic indicator for the progression of cancer, recurrence, metastatic spread, and survival. Thus, HA and its metabolic pathways may be considered as prospective aims for anticancer medication.

## Figures and Tables

**Table 1:** HAS expression-dependent effects on cancer type.

HAS Type	Cancer Type	Effect of Upregulation	Effect of Downregulation
*HAS1*	Breast cancer (in vitro)	-	↓ cancer development and metastasis [105]
	Melanoma (ex vivo)	-	adverse prognosis [101]
	Bladder cancer (ex vivo)	↑ disease progression, recurrence, adverse prognosis	-
*HAS2*	Breast cancer (in vitro)	↑ invasion of breast cancer cells by decreasing tissue metalloproteinase inhibitor 1 (TIMP-1) [104].	↑ chemotherapy sensitivity ↓ tumor cell migration and proliferation [105,106].
	Osteosarcoma (in vitro)	-	↓ tumor cell migration and proliferation [110]
	Prostate cancer (in vivo)	↑ angiogenesis [103]	-
	Colon cancer (in vitro)	-	↓ cells’ ability to spread ↓ matrix metalloproteinase-7 (MMP-7) expression and activity [109]
	Melanoma (ex vivo)	-	adverse prognosis [101]
*HAS3*	Breast cancer (in vitro)	-	↓ cancer development and metastasis [105]
	Prostate cancer (in vitro)	↑ proliferation, extracellular matrix deposition [111]	-
	Colon cancer (in vitro)	-	↓ cells’ ability to spread [109]

**Table 2:** HYAL expression-dependent effects on cancer type.

HYAL Type	Cancer Type	Effect of Upregulation	Effect of Downregulation
*HYAL-1*	Prostate cancer (xenografts)	↑ lung metastases	↓ tumor development ↓ capacity to metastasize by triggering cell cycle arrest in the G2/M phase [116]
	Breast cancer (in vitro)	↑ Cell proliferation, migration, invasion, and angiogenesis [118,119]	-
	Bladder cancer (in vitro, xenograft)	↑ invasiveness ↑ tumor growth rate [120]	↓ tumor development ↓ capacity to metastasize by triggering cell cycle arrest in the G2/M phase [116]
	Colon cancer (in vitro and in vivo)	↓ growth rate of tumor cells [121]	-
	Pancreatic ductal adenocarcinoma (in vivo)	-	poorer prognosis [125]
	Ovarian cancer (ex vivo)	-	poorer prognosis [126]
	Endometrial cancer (ex vivo)	-	poorer prognosis [127]
*HYAL-2*	Lung cancer (in vivo, mice)	↓ tumor development [124]	-
	Pancreatic ductal adenocarcinoma (in vivo)	-	poorer prognosis [125]
	Ovarian cancer (ex vivo)	-	poorer prognosis [126]
	Endometrial cancer (ex vivo)	-	poorer prognosis [127]

## Data Availability

Not applicable.
